# The potential of omics approaches to elucidate mechanisms of biodiesel-induced pulmonary toxicity

**DOI:** 10.1186/s12989-018-0284-y

**Published:** 2019-01-08

**Authors:** Liza Selley, David H. Phillips, Ian Mudway

**Affiliations:** 1 MRC Toxicology Unit, University of Cambridge, Hodgkin Building, Lancaster Road, Leicester, LE1 9HN UK; 2 Department of Analytical, Environmental and Forensic Sciences, MRC-PHE Centre for Environment & Health, School of Population Health and Environmental Sciences, Franklin-Wilkins Building, King’s College London, London, SE1 9NH UK; 3 NIHR HPRU in Health Impact of Environmental Hazards, Franklin-Wilkins Building, King’s College London, London, SE1 9NH UK

**Keywords:** Biodiesel, Pulmonary toxicity, Transcriptomics, Metabolomics, Hypothesis generation, Mechanism

## Abstract

**Background:**

Combustion of biodiesels in place of fossil diesel (FD) has been proposed as a method of reducing transport-related toxic emissions in Europe. While biodiesel exhaust (BDE) contains fewer hydrocarbons, total particulates and carbon monoxide than FD exhaust (FDE), its high nitrogen oxide and ultrafine particle content may still promote pulmonary pathophysiologies.

**Main body:**

Using a complement of in vitro and in vivo studies, this review documents progress in our understanding of pulmonary responses to BDE exposure. Focusing initially on hypothesis-driven, targeted analyses, the merits and limitations of comparing BDE-induced responses to those caused by FDE exposure are discussed within the contexts of policy making and exploration of toxicity mechanisms. The introduction and progression of omics-led workflows are also discussed, summarising the novel insights into mechanisms of BDE-induced toxicity that they have uncovered. Finally, options for the expansion of BDE-related omics screens are explored, focusing on the mechanistic relevance of metabolomic profiling and offering rationale for expansion beyond classical models of pulmonary exposure.

**Conclusion:**

Together, these discussions suggest that molecular profiling methods have identified mechanistically informative, novel and fuel-specific signatures of pulmonary responses to biodiesel exhaust exposure that would have been difficult to detect using traditional, hypothesis driven approaches alone.

## Background

In 2017, UK road users travelled 325.5 billion miles [1]; a figure that is forecast to increase by 75 billion by 2040 [2] and follow a trend that is being predicted globally [3]. Fossil fuel combustion contributes significantly to traffic-related air pollution, releasing pollutant gases (including nitrogen oxides (NO_x_), sulphur dioxides and carbon monoxide (CO)) and particulates [4]. As these composition derived particles are of respirable size [5–7], exist ubiquitously in urban areas and have been shown to induce inflammatory pulmonary pathophysiologies [8–11], regulators welcome interventions that reduce their emission.

Biodiesel is a liquid fuel produced through transesterification of vegetable oils, animal fats or waste cooking oil. This base-catalysed reaction is driven by adding alcohol to the feedstock [12], converting the parent product to its less viscous alkyl-ester. When blended (up to 20% volume) with fossil diesels (FD), biodiesels can power modern compression-ignition engines efficiently without demanding modified transport or storage measures [13, 14].

Biodiesel has a higher oxygen content than FD, encouraging more complete fuel combustion [13]. Coupled with an absence of aromatic and sulphurous compounds, the resulting emissions can contain 90% fewer unburned hydrocarbons, 75–90% fewer polycyclic aromatic hydrocarbons (PAHs), 43% less CO and 55% less particulate matter (PM) than FD exhaust (FDE) [15, 16], depending on the engine and combustion conditions used to burn the fuel. As these pollutants associate significantly with the incidence of airway infection and respiratory symptoms, onset and exacerbation of inflammatory pulmonary diseases and rates of respiratory hospital admissions in heavily trafficked areas [17–20], biodiesel usage has been encouraged in Europe, with the 2009/28/EC directive striving to replace 10% of transport fuel with biodiesel by 2020 [21].

Despite some improvements in emission profiles, BDE generally contains more NO_x_ and ultrafine particles (PM_0.1_) than FDE [22] with particle emissions increasing with the chain length and degree of double bond saturation in the feedstock fatty acids [23]. Given that PM_0.1_ have been shown to exhibit greater inflammatory potential and oxidant capacity than larger particles [24, 25] and that both PM_0.1_ and NO_x_ have been associated with mortality from respiratory diseases [26, 27], it is imperative that the impacts that biodiesel exhaust (BDE) exposure have on pulmonary health are characterised and communicated to policymakers. Furthermore, BDE contains a number of molecules that have the potential to induce pulmonary toxicity that are not found in FDE (or are present at significantly lower levels). Esters such as those found within BD can produce toxic, mutagenic and carcinogenic carbonyls when burnt in the presence of O_2_, with concentrations of formaldehyde, acetaldehyde, acrolein, acetone, propionaldehyde and butyraldehyde increasing linearly as the BD component of blends increases [28]. Additionally, BD fuel esters themselves have been detected in BDE, including several species that are known to induce pulmonary irritation [29].

Two complementary approaches have been employed to investigate particulate toxicity; traditional hypothesis-driven methods and, more recently, agnostic approaches that employ various untargeted omics platforms to identify global patterns of response to generate novel insights and new hypotheses. Traditional approaches are of considerable value to epidemiologists because they associate pathophysiological endpoints (including airway responsiveness and respiratory symptoms) with underlying molecular events, i.e. they support causal inference. Furthermore, with BD being proposed as a replacement for FD, they enable investigation of endpoints that have previously been validated as markers of particle toxicity allowing comparative toxicological assessment. The techniques are however, limited in their ability to uncover novel pathways, making them less informative for mechanistic studies of complex aerosols with novel constituents.

**Fig. 1:**
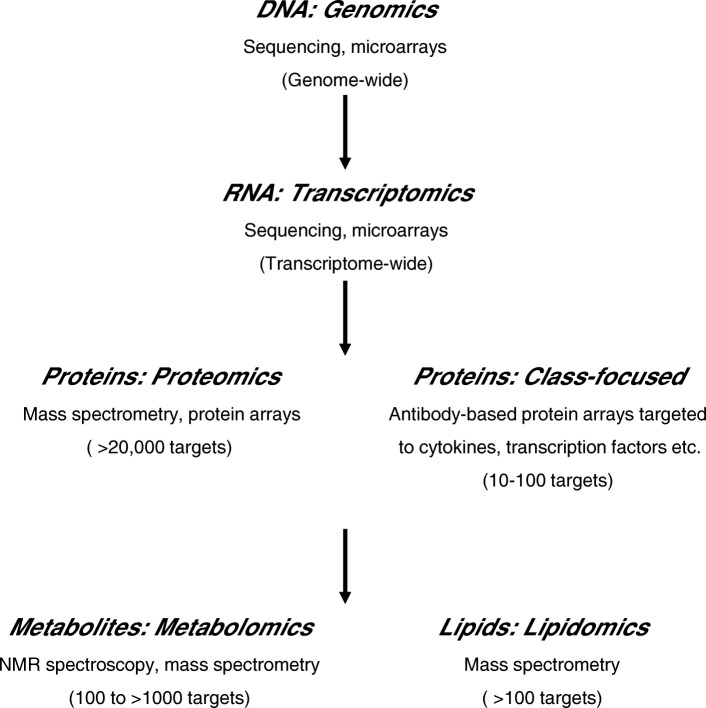
Major techniques and technologies employed during molecular profiling. Typical numbers of molecules detectable by each platform (during a full platform analysis) are displayed in brackets

In contrast, high-throughput omics techniques characterise the expression of 100s–1000s of molecules, or 10s of thousands of transcripts, simultaneously, enabling additional molecular events to be tested for association with exposures in an untargeted manner (Fig. 1). This approach has significantly advanced characterisations of FDE toxicity, demonstrating, for example, that lung cells undergo a hierarchical oxidative stress response following exposure [30] and that impairment of macrophage phagocytic functions may be influenced by differential expression of genes implicated in cytoskeletal rearrangements [31]. Still less commonplace than the cheaper traditional techniques, omics screens are gaining popularity as methods of characterising pulmonary responses to BDE exposure.

The information that hypothesis-driven studies have provided on biodiesel-induced pulmonary toxicity has been reviewed comprehensively [32, 33]. Following from this work, our review focuses on recent transitions towards omics-based assessments of BDE-induced pulmonary toxicity. The work aims to evaluate the effectiveness of including molecular screens in characterisations of pulmonary toxicity mechanisms and to comment on the usefulness of these data in terms of understanding whether BD blends are a ‘healthy’ replacement for pure FD. To produce these evaluations, studies of biodiesel toxicity were identified within the PubMed database (http://www.ncbi.nlm.nih.gov/sites/entrez) using the key words ‘biodiesel AND lung OR pulmonary’ combined with ‘toxicity/ inflammation/ genotoxicity/ transcriptomics/ mass spectrometry or omics’. Reports from human cohort studies, animal exposures or in vitro modelling were included for review while studies performed in plants or with raw biofuels were excluded from the analysis.

## Main body

### Hypothesis-driven approaches to exhaust toxicity

The London smog episode in 1952 was one of the events that initiated research into the toxicity of ambient pollution, and since that time, characterisation of pulmonary responses to inhaled xenobiotics has advanced greatly [34]. Much of our knowledge of the adverse effects that exhaust emissions exert upon the lung is derived from human experimental chamber studies of FDE exposure [35, 36], or exposures of individuals to micro-environments with high traffic related emissions [37–39]. Historically, these studies have sought to measure validated markers of pulmonary responses in a targeted manner and are supported by increasing evidence from the epidemiological literature that respiratory mortality and morbidity is strongly related to components within the airshed that reflect emissions from diesel exhaust, such as elemental and black carbon [40–42].

Experimental chamber studies that have examined responses to the full diesel aerosol: particulates, volatile organic species and co-pollutant gases (NO2, NOx and CO) have shown that diesel exhaust (100–300 µg/m^3^ PM_10_) breathed over short durations of 1–2 h (in individuals performing intermittent exercise) can elicit the full pathway of neutrophilic inflammation in the lung [39], from upregulation of endothelial adhesion molecules [39], expression of pro-inflammatory cytokines [39], such as IL-6 and Gro-α, to established airway neutrophilia in both the airway tissues and respiratory tract lining fluids [39]. These responses occur in parallel with increased mast cell numbers in the bronchial mucosa and lymphocytosis [39, 43].

In these studies, largely conducted on young heathy volunteers, the causal role of the combustion derived nanoparticles in respiratory responses have been established experimentally using particle traps [44]. In addition, experiments comparing engines running under idling versus under load (standard city cycle), which impacts on the relative proportion of elemental carbon to organic carbon, have yielded broadly similar results, although some evidence of lower airway eosinophilia was noted followed exposure to diesel exhaust from the engine under load. Underpinning these inflammatory responses in the airways, activation of redox sensitive pathways has been reported, most clearly through the increased nuclear localisation of nuclear factor kappa-light-chain-enhancer of activated B cells (NFκB) and activator protein 1 (AP-1) [45], but also through the upregulation of the epidermal growth factor receptor (EGRF) in the bronchial epithelium [46].

Less clear, until very recently, has been the mechanism by which diesel exhaust could lead to an exacerbation of asthma symptoms. Early studies, simply looking at the inflammatory response of mild and moderate asthmatic exposures to diesel failed to demonstrate clear evidence of augmented allergic inflammation [36], contrasting with the evidence of upper airway inflammation and pulmonary irritation (measured as a sustained reduction in forced expiratory volume) seen in asthmatics exposed to high levels of FDE as they walked along Oxford Street, London, UK [39]. Some mechanistic basis for increased respiratory symptoms in response to diesel particulates has recently been reported by experimental evidence demonstrating that the organic constituents associated with diesel particles can activate airway sensory afferents; a response that could be inhibited using a transient receptor potential ankyrin-1 antagonist, provision of antioxidants (N-acetyl cysteine) and inhibition of the aryl hydrocarbon receptor (AhR) [46]. Additionally, work by Carlsten et al. has demonstrated that diesel challenge of allergic individuals appears to sensitise the airways to subsequent allergen challenge [47, 48]. Thus, collectively over the last 20 years, the evidence base that FDE can impact adversely on both the healthy and allergic airway has increased and constitutes a substantial mechanistic underpinning of the epidemiological findings of adverse health effects associated with exposure to traffic-derived pollutants, especially FDE [49].

### Biodiesel toxicity: the influence of fossil diesel studies

A decade ago, few groups had explored the health impacts of BDE exposure; either from an epidemiological or mechanistic viewpoint and unlike for FDE, human exposure studies had not been performed. However, histological changes such as increases in the lung: body ratios had been observed in female rats that had been exposed repeatedly to 0.5 mg particles /m^3^ of 100% soybean BDE via inhalation for thirteen weeks, along with dose-dependent increases in pulmonary macrophage counts and particle loading at lower and intermediate doses (0.04 and 0.2 mg/m^3^) [50]. These changes were consistent with the development of inflammatory pulmonary lesions and bronchiolar metaplasia in the alveoli where macrophages remained particle-laden for 28 days [50]. In terms of cytotoxicity, the soluble organic fraction (SOF) of rapeseed methyl ester (RME) exhaust was identified as more potent than its FD counterpart when applied to L929 mouse fibroblasts; while in the Ames test, the SOF of FDE-PM was more mutagenic to bacteria than BDE-derived particulate matter (BDE-PM) [32, 51]. Mutagenicity tests were also performed on rat hepatocytes, indicating that differences in the mutagenic potential of FDE and BDE were less pronounced in mammalian cells compared to bacteria; potentially reflecting differences in their metabolic capabilities [51]. Taken together it was proposed that studies of BDE toxicity should continue to draw from characterisations of FDE toxicity [52]. Indeed, comparisons between the toxicological potential of alternative and existing products are common-place in many fields, [53] and most biodiesel-themed studies have employed this approach.

While studies have continued to explore the genotoxicity of BDE, relative to FDE in vitro, a comprehensive evaluation of carcinogenicity of BDE is not currently possible because of a lack of human exposure studies and animal bioassays. In vitro investigations have provided mechanistic associations between components of FDE and the mutagenic and genotoxic effects of FDE exposure, but the classification of FD emissions as carcinogenic to humans (Group 1) by the International Agency for Research on Cancer was enabled by sufficient evidence being available from epidemiological studies and animal carcinogenicity assays [54].

Regardless of the type of diesel fuel, genotoxicity is mainly associated with the particulate fraction [32]. In an early study, comparison of organic extracts of a BDE with a FDE found that the former had approximately half the bacterial mutagenic activity of the latter, a reduction that correlated with decreased concentrations of PAHs and nitro-PAHs [55]. Some, but not all, combinations of fuel and engine type result in lower concentrations of particulate emission for BD or BD blends than for standard FD fuels, accompanied by lower bacterial mutagenicity [32]. Much of the mutagenic activity is associated with PAHs and with nitro-PAHs. Under oxidative or nitro-reductive conditions, organic extracts of FD and BD emissions generate DNA adducts in vitro, with properties compatible with those of adducts formed by PAHs and nitro-PAHs [56]. In one study, adduct formation by a BD blend was ~ 50% of that of conventional diesel [56], but in another, DNA adduct formation by extracts of particulates from rapeseed oil combustion emissions was comparable to that of conventional diesel emissions [57].

When generated from a diesel engine fitted with a catalyst, an RME BDE, but not a hydrogenated vegetable oil (HVO) BDE had lower particulates than a FDE and lower genotoxicity, measured by the comet assay, in RAW 264.7 macrophages [58]. This was accompanied by lower formation of reactive oxygen species (ROS). Not all studies have found lower activity than BDE than with FDE. A comparison between BDE and FDE from light-duty engines found similar ROS production and comet formation in A459 cells [59]. When organic extracts of particle emissions of different biodiesel blends were tested in human bronchial epithelial BEAS-2B cells, the frequency of micronucleus formation was similar for neat biodiesel, neat diesel and a 30:70 blend of the two [60]. In an in vivo study in rats, inhalation exposure resulted in induction of detectable levels of bulky DNA adducts in the lungs in the case of FDE, but not with BDE, but differences in levels of 8-oxodGuo in DNA by the two emissions sources were not observed [61].

Consistent with the notion that FDE stimulates inflammatory and oxidative stress responses in pulmonary cells [62], it was demonstrated that the SOF of soybean alkyl ester PM was a more potent inducer of IL-6 and IL-8 secretion than the equivalent extract of FDE-PM in BEAS-2B bronchial epithelial cells [63]. This observation was supported by data demonstrating that emissions produced from a 50% RME blend also induced greater cytotoxicity and IL-6 release than FD-derived counterparts in BEAS-2B cells, even in the presence of a DPF. Interestingly, the authors of this study demonstrated that their results were dependent on the data being normalised to distance driven during sample collection, indicating that rate of particle emission is an important metric to consider during dosing [64]. In THP-1 monocyte-derived macrophages, PM from B20 soy methyl ester (SME) induced stronger toxicity than FDE-PM, as determined by quantification of ROS 1-2 h post-exposure [29]. Similarly, particles produced from HVO stimulated a more potent dose-dependent increase in TNF-α and MIP-2 secretion than FDE-PM in RAW 264.7 cells, reaching statistical significance after exposure to 50 μg/ml doses rather than 150 μg/ml. While RME particles also induced dose-dependent increases in TNF- α and MIP-2 secretion, the response was significantly less than for FDE-PM or SME BD [58] indicating variable inflammatory potency for BD produced from different feedstocks.

Regarding the ranking of FD and BD potency, considerable inter-study discrepancies have been observed in vivo. In a murine model, Yanamala et al. observed that PM from pure fatty acid methyl ester (FAME) emissions induced cellular infiltration into the lung to a significantly greater extent than FDE-PM. This inflammatory response was characterised by neutrophilia 1-day post-exposure, followed by macrophage influx at the 7-day timepoint. In addition, reductions in pulmonary cell viability were observed at an earlier timepoint after BD exposure (1 day, compared with 7 days for FD) and markers of oxidative damage (4-hydroxynonenal protein carbonyls) and remained visible for a longer period (28 days) [65]. While Fukagawa et al. also reported that mice exposed to PM from a B20 SME blend had greater levels of protein carbonyls in their lungs than mice exposed to FDE-PM, they did not detect fuel-dependent differences in cellular infiltration with the airways, as assessed by bronchoalveolar lavage [29]. In further contrast, studies performed by the U.S Environmental Protection Agency (EPA) found FDE-PM exposures to be more toxic than BDE-PM exposures, with mice exposed to FDE-PM for 4 h exhibiting an 8-fold increase in pulmonary neutrophil influx and MIP-2 secretion than mice exposed to PM_2.5_ from B20 or B100 SME blends (at 500 μg/m^3^ doses only) [66]. Using a model of Wistar Kyoto rats, the EPA also demonstrated that FDE induced greater pulmonary injury (as determined by increased BALF GGT activity) than BDE (100% or 20% blend) following 24 h exposures and increased neutrophilia after dosing for 4 weeks [67]. As with their murine study [66], these effects were only visible at the highest tested dose (500 μg/m^3^) [67] so may not be especially representative of real-world exposures which are estimated to fall between 15 and 20 μg/m^3^ for urban roadside PM_2..5_ [68]_._ Despite this, it may be useful to note that FDE-PM but not PM from B20 or B100 SME blends increased proliferation of resting peribronchiolar lymph node cells and production of T helper cell cytokines in a murine model of allergic inflammation after 4 weeks of repeated exposure at 500 μg/m^3^ doses [39]. Such data demonstrate the possibility that BDE is less likely than FDE to induce allergic responses in individuals with allergic airway conditions.

Both in vitro and in vivo, these inter-study discrepancies have been attributed to differences in the fraction of exhaust tested. In human alveolar epithelial cells, apoptosis, viability and inflammatory responses have all been shown to be altered adversely by the presence of exhaust gases, [69] with semi-volatile extracts reducing viability to a greater extent than PM in the case of palm FAME [70]. The physicochemical properties of particles have also been considered, with particle surface area, transition metal content and mass concentration and diameter all being shown to contribute to inflammatory and cytotoxic potential in vitro [69] [71]. Such parameters may vary with choice of engine type, percentage blend of fuels [70], inclusion of emission modifying technologies [58, 64] and driving conditions [72]; factors that can introduce inter-study differences to both the BDE and FDE samples. Additionally, the choice of feedstocks may influence BDE-PM characteristics considerably; fuels derived from waste cooking oil for example, are chemically distinct to those produced from raw vegetable oils due to the array of thermolytic reactions that modify the feedstock during heating [73]. Furthermore, pollutant samples produced by splash blending of pure fuels to achieve B20 and B50 blends (as employed in the EPA studies) tend to exhibit linear increases in BD-specific chemical components as BD content increases while commercially available blends do not [33]. While these linear relationships may assist relation of toxicological endpoints to specific emission components, it is important to consider that splash-blended fuels can contain novel components [33], thus PM from splash blended B20 could cause different biological effects to PM from commercial B20.

Adding to variation between in vivo analyses, are possibilities of species-dependent threshold effects. Rodent models of particle exposure are susceptible to pulmonary overload (a progressive reduction in particle clearance caused by loss of motility in alveolar macrophages that results in chronic inflammation, fibrosis and tumorigenesis [74]) as well as minimal effective dose thresholds for particle-induced inflammatory responses [75]. Choice of exposure methods for rodent studies can impact on both the dose-rate (tissue-wide and compartmentally specific) and clearance or particles within the lung; both factors that can influence the applicability of threshold effects to individual models. As an example, intratracheal instillation of TiO_2_ particles (especially within the ultrafine fraction) induces significantly greater cellular infiltration of BALF than inhalation of the same particles, partly due to the total intended dose being administered all at once, rather than across an extended period [76]. Based on exposure-response curve data covering non-interventional exposures to PM doses as low as 2 μg/m^3^, and observations of low rates of tumour development in chronically particle-exposed individuals, it has been suggested that these thresholds are not relevant for humans [75, 77]. This raises additional questions as to whether data from rodent models that explore particle potency can be applied simplistically to human exposure scenarios or compared reliably with results from human cohort studies. Regardless of the components that drive these inter-study differences, their existence highlights the necessity to consider outcomes of BDE exposure as absolute values. While comparisons between BDE and FDE toxicity assist decision-making regarding the option of replacing fossil fuels with renewable alternatives, very few studies have concluded that BDE exposure has no adverse effects on pulmonary health or function. As such, absolute values for BDE toxicity metrics may prove vitally informative for policymakers, indicating the maximum levels of exposure that can be tolerated by humans. Additionally, greater attention should be paid to the metrics used to determine particle dosing. Across the field of emissions toxicology, convenience and desire for comparability with historical studies has encouraged the widespread use of particle mass for this purpose. Increasingly however, surface area has been argued to relate better to particle-induced responses, including influx of immune cells and cytokine secretion in mice [75] and pulmonary overload in rats [74]. Importantly, the heightened inflammatory potential reported with PM_0.1_ compared with larger particles of equivalent composition, has been shown to disappear when exposure doses are equalised for surface area, rather than mass [78]. Given that BDE contains considerably greater proportions of PM_0.1_ than FDE, it may be prudent to consider surface area as a dose metric for future comparisons of toxicity.

### Exploration of effector pathways

The contributions of prototypical inflammatory pathways to PM-induced responses have been studied widely in models of FDE-PM exposure. Both aromatic and polar fractions of FDE-PM have been shown to induce nuclear receptor factor 2 (Nrf2)-dependent heme oxygenase-1 (HO-1) expression, activation of mitogen-activated protein kinase (MAPK) pathways and induction of NFκB signalling in macrophages and epithelial cells [79, 80]. Similarly, repeat exposure to BDE-PM (produced through combustion of a FD-BD blend) promoted oxidative stress and inflammation in mice, as evidenced by increased concentrations of macrophages in the lungs, and heightened expression of TNF-α, NFκB, Nrf2 and HO-1 proteins [81]. Additionally, it was shown that PM produced through combustion of 20% blends of waste-grease biodiesel (WGPM) stimulated greater extracellular signal-regulated kinase (ERK)1/2 phosphorylation in BEAS-2B cells than FDE-PM [71]. These studies indicate that BDE-PM-induced responses share features with other PM-induced responses in redox sensitive signally pathways, as demonstrated through ERK1/2 activation, can be stimulated with differential potency compared with FDE-PM [71].

Focus has also moved towards characterisation of the mechanisms by which BD-induced pathways are stimulated in the lung. PM of varying composition have been shown to induce airway inflammation via activation of toll-like receptors (TLR); pattern recognition receptors that stimulate cytokine secretion, via NFκB, activating protein-1 and MAPK driven pathways [82]. FDE-PM have been shown to induce cytokine secretion and neutrophilia in a TLR4-dependent manner [83], but composition-dependent differences (including metal and endotoxin content) have been observed in the range of TLR species with which particles interact [84], indicating that this mechanism may differ for BDE-PM. Indeed, Traviss et al. demonstrated that WGPM exposure increased expression of TLR2, but not TLR4 in THP-1 macrophage lysates while FDE-PM displayed little association with TLR2 expression [71].

In addition, combustion products have been shown to trigger pulmonary responses by activating dormant cytosolic AhRs. Once bound by PAHs from the surface of combustion particles, AhRs undergo a conformational change that permits their translocation into the nucleus. In terms of PM toxicity, a widely explored outcome of this activity is induction of cytochrome P450 (CYP) enzymes, which play a critical role in the oxidative metabolism of lipophilic compounds, including PAHs themselves. While chemically inert in their environmental form, PAHs display considerable redox activity once oxidised by CYPs, promoting development of oxidative stress and mutagenicity [85].

Unfiltered combustion products of a B99 blend of BD have been shown to significantly upregulate *CYP1A1* expression in human bronchial epithelial cells after 60 min of exposure to fresh, pure exhaust. Unfiltered FD combustion products also enhanced *CYP1A1* expression, producing a statistically significant (but numerically comparable) increase at a faster rate than the BDE (20 min). For both fuel types, emissions collected in the presence of diesel particulate filters induced considerably greater *CYP1A1* expression than their unfiltered counterparts, demonstrating that the gas and semi-volatile components of both BDE and FDE contribute greatly to their toxicity [86] Many in vitro studies of exhaust toxicity (and indeed, technical assessments of engine emissions) employ particle mass, number or surface area as a means of determining exposure dosages, with surface area suggested to be most relevant for assessments of pulmonary inflammation [87]. However, the evidence that volatile exhaust components can regulate critical aspects of the pulmonary response to PAH exposure indicates that particle-focused dose metrics may not represent the exposure-response relationship for total exhaust very well.

The effects that feedstock compositions have on BDE-induced *CYP1A1* expression have also been explored in bronchial epithelial cells, demonstrating that PM produced from a blend of 13% HVO and 7% FAME in FD induces *CYP1A1* expression to a greater extent than PM produced through combustion of non-supplemented FAME and FD blends (both 7 and 20%) after both 4 and 20 h of exposure [88]. This trend was observed consistently across a dose range of 10–100 μg/ml, but occurred in contrast to total PAH contents for the particles, indicating that specific PAH combinations or non-PAH components contributed to *CYP1A1* expression in this model.

Focusing further on mechanisms of BD-induced CYP activation, H441 human pneumocytes were used to investigate the potential involvement of transient receptor potential ankyrin 1 (TRPA1) ion channels (mechanistic contributors to PM-induced disruption of calcium homeostasis and downstream cytokine secretion). The data showed that while *CYP1A1/1B1* over-expression was induced BDE-PM in a dose-dependent manner (and with far greater potency than with FDE-PM), it was not attenuated by TRPA1 antagonists. While this indicated a lack of relationship between TRPA1 activity and *CYP* expression, IL-8 secretion did decrease significantly (~ 60%) in the presence of the antagonist, highlighting a role for TRPA1 in stimulation of BDE-PM-induced inflammatory cascades [89].

### Progression towards a global approach

#### Cytokine arrays

By using molecular profiling methodologies, it is possible to obtain data with both qualitative and quantitative properties. Thus, through the assessment of large panels of variables, molecular interactions can be explored as part of a wider network, to determine which molecules are induced, or inhibited across the time-course of a response. Given that development and resolution of pulmonary inflammation are regulated by the complex interplay of pro and anti-inflammatory mediators, application of profiling approaches to the context of BDE exposure was led by the performance of cytokine arrays (summarised in Table 1). While continuing to compare the effects of exposure to BDE with those of FD emissions, these studies enabled novel hypotheses to be drawn regarding fuel-specific mechanisms of inflammatory cell recruitment to the lungs, thus offering policymakers an additional layer of evidence to support or oppose BD usage.

**Table 1:** Documented cytokine secretion profiles for pulmonary cells or tissues following exposure to biodiesel emissions

Study	Malorni et al. 2017 [92]	Fukagawa et al. 2013 [29]				Yanamala et al. 2013 [65]		Shvedova et al. 2013 [90]
Exposure	20% FAME PM_0.1_ (organic fraction)	20% SME Total PM				100% FAME Total PM		100% SME Total PM
Model	A549	THP-1	BEAS-2B	Mouse lung	Mouse BALF	Mouse lung	Mouse BALF	Mouse Lung
bFGF	**↓**	**–**	**–**	**N**	**N**	**–**	**–**	**N**
CCL5 (RANTES)	**N**	**N**	**N**	**N**	**N**	**↑**	**↑**	**N**
CXCL1 (KC)	**N**	**N**	**N**	**–**	**–**	**↑**	**↑**	**N**
CXCL9 (MIG)	**N**	**N**	**N**	**N**	**N**	**↑**	**↑**	**N**
Eotaxin	**↓**	**–**	**–**	**N**	**N**	**↑**	**–**	**N**
G-CSF	**↓**	**↑**	**–**	**↑**	**↑**	**↑**	**↑**	**N**
GM-CSF	**–**	**–**	**–**	**–**	**–**	**–**	**–**	**N**
IL-10	**–**	**–**	**–**	**–**	**–**	**–**	**–**	**↑**
IL-12p40	**–**	**–**	**–**	**N**	**N**	**↑**	**↑**	**N**
IL-12p70	**–**	**–**	**–**	**–**	**–**	**↑**	**↑**	**↑**
IL-13	**–**	**–**	**–**	**–**	**–**	**–**	**–**	**N**
IL-15	**↓**	**–**	**–**	**–**	**–**	**–**	**–**	**N**
IL-17	**↓**	**–**	**–**	**–**	**–**	**–**	**–**	**N**
IL-18	**N**	**N**	**N**	**N**	**N**	**–**	**–**	**N**
IL-1α	**N**	**N**	**N**	**–**	**–**	**↑**	**↑**	**N**
IL-1β	**–**	**–**	**–**	**–**	**–**	**↑**	**↑**	**N**
IL-1ra	**–**	**–**	**–**	**N**	**N**	**N**	**N**	**N**
IL-2	**↓**	**–**	**–**	**–**	**–**	**–**	**–**	**N**
IL-3	**N**	**N**	**N**	**N**	**N**	**–**	**–**	**N**
IL-4	**–**	**–**	**–**	**–**	**–**	**↑**	**↑**	**N**
IL-5	**–**	**–**	**–**	**–**	**–**	**–**	**–**	**N**
IL-6	**↓**	**–**	**–**	**↑**	**↑**	**↑**	**↑**	**↑**
IL-7	**–**	**–**	**–**	**–**	**–**	**N**	**N**	**N**
IL-8	**–**	**–**	**↑**	**N**	**N**	**N**	**N**	**N**
IL-9	**–**	**–**	**–**	**–**	**–**	**–**	**–**	**N**
IFNγ	**–**	**–**	**–**	**–**	**–**	**–**	**↑**	**–**
IP-10	**↓**	**–**	**–**	**↑**	**↑**	**–**	**–**	**N**
LIF	**N**	**N**	**N**	**N**	**N**	**↑**	**–**	**N**
MCP-1	**–**	**–**	**↑**	**–**	**–**	**↑**	**↑**	**↑**
M-CSF	**N**	**N**	**N**	**–**	**–**	**–**	**–**	**N**
MIP-1α	**–**	**–**	**–**	**–**	**–**	**↑**	**↑**	**N**
MIP-1β	**–**	**–**	**–**	**N**	**N**	**↑**	**↑**	**N**
MIP-2	**N**	**N**	**N**	**N**	**N**	**↑**	**–**	**N**
PDGF	**↓**	**–**	**–**	**N**	**N**	**↑**	**–**	**N**
TNF-α	**–**	**–**	**–**	**–**	**–**	**–**	**↑**	**↑**
VEGF	**–**	**–**	**–**	**N**	**N**	**–**	**–**	**N**

**↑** represents statistically significant increase in secretion compared with particle-free controls while **↓** represents statistically significant decreases in secretions. **–** demonstrates no statistically significant change in secretion compared with non-exposed controls and **N** indicates instances where a cytokine species was not measured. Basic Fibroblast growth factor (bFGF), Chemokine (C-C-motif) ligand (CCL), Chemokine (C-X-C motif) ligand (CXCL), Granulocyte-colony stimulating factor (G-CSF), Interferon-γ neutralising (IFNγ), Interferon-γ-induced protein 10 (IP-10), Leukaemia inhibitory factor (LIF), Monocyte chemoattractant protein-1 (MCP-1), Macrophage colony stimulating factor (M-CSF), Macrophage inflammatory protein (MIP), Platelet-derived growth factor (PDGF), Tumour necrosis factor-α (TNF-α), Vascular endothelial growth factor (VEGF)

Performing an early, small-scale cytometric array on lung tissue, Shvedova et al. observed differential patterns of cytokine expression in the lungs of mice exposed to 0–500 μg/m^3^ concentrations of SME exhaust or FDE for a 4-week period [90]. The authors noted that BDE stimulated MCP-1 secretion by 38% more than FDE and that this response was consistent with Finch et al.’s observation that repeated exposure to BDE stimulated macrophage recruitment to rodent lungs [50]. As this response occurred in parallel to increased expression of IL-12p40, in the absence of an IFNγ, response with BDE exposure, the group hypothesised that BDE (but not necessarily FDE), induced macrophage activation via non-classical pathways. They also demonstrated that both exposure types induced increases in cytokine accumulation in the liver but that these effects were less pronounced than those observed in the lungs [90].

Several larger array platforms have been adopted to explore BDE and FDE toxicity, offering the distinct advantage of a more comprehensive qualitative range. Indeed, Yanamala et al. detected significant elevations of 17 cytokines and chemokines in murine pulmonary tissue following acute exposure to an 18 μg/mouse bolus of FAME exhaust PM (B100); a signature that translated closely to bronchoalveolar lavage fluid (BALF) [65] and was concordant with the induction of IL-12p70, IL-6, MCP-1 and TNFα secretion that was reported by Shvedova et al’s smaller scale SME study (Table 1). Yanamala et al. also observed evidence that BDE-PM induced secretion of ten cytokines and chemokines acutely, whilst FDE-PM did not [65]. This indicated that BDE-PM induces a more complex inflammatory cascade than FDE-PM; with the authors suggesting potential contributions of allergic inflammation and type 2 T helper cell responses [65]. Furthermore, the study demonstrated that IL-1a and IL-12p40 concentrations remained elevated 7 and 28 days after inoculation with BDE-PM, but not FDE-PM, indicating that BDE promotes a more prolonged pulmonary response [65].

Fukagawa et al. [29] used cytokine arrays to explore the effects of exposure to exhaust from commercially relevant B20 BD blends. Analysing the pulmonary tissue and BALF of mice, the authors demonstrated that repeated exposure to combustion particles from B20 SME (84 μg/ treatment over 3 days) induced pulmonary secretion of G-CSF, IP-10 and IL-6. While variations in study design and sample collection prevent a simple comparison of these results with those of Shvedova et al.’s murine study of exposure to PM from 100% SME [90], the authors did demonstrate that FDE-PM did not alter pulmonary cytokine profiles when compared with controls, despite inducing comparable BALF cell profiles to the B20 sample. This observation illustrates the value of board-based molecular investigations to comprehensively characterise the impacts that BDE-specific components have on pulmonary cells and is supported by the group’s observations in vitro. Using THP-1 and BEAS-2B cell lines, the group demonstrated that B20 BDE-PM and FDE-PM induce differential responses to one another in both macrophages and bronchial epithelial cells. A major concern for assessments of pollutant inflammogenicity is the ability of carbonaceous particles to bind cytokines, reducing the effective concentration during processing, thus risking introduction of type II errors during cytokine/chemokine quantifications [91]. Importantly, Fukagawa et al. used a cell-free system to demonstrate that neither particle species altered the concentration of cytokine standards, indicating that the observations were products of true interactions between the particles and the cells [29] rather than artefacts of differential particle composition.

Evidence that the physicochemical properties of particulates influence their toxicity was reported by Malorni et al. who demonstrated that particles with an aerodynamic diameter of less than 10 nm from either B20 FAME or pure FD emissions (1.2 or 4 ppm) inhibited cytokine secretion globally in A549 epithelial cells, [92]. Based on data collected for 27 targets (Table 1), the authors suggest that the small size of the particles was responsible for this, facilitating interference with mechanisms of protein synthesis or secretion, alternatively, that the assay had captured negative feedback loops following initial elevations in cytokine secretion [92]. It should however be noted that A549 express unusually high levels of HO-1 and Nrf2 [93] and that cells cultured under standard laboratory conditions experience a high basal layer of oxidative stress [94] so may respond differentially to oxidative stimuli compared with cells in vivo. Once again, the authors report biodiesel-specific alterations to the cellular cytokine profile.

#### Transcriptomics

To characterise a mild inflammatory response in the lungs of rats exposed to 20% blends of RME BDE for 7 or 28 days, the expression of 32 genes in pulmonary tissue was measured [95]. This semi-targeted approach drew upon the multivariate nature of transcriptomic analysis, but focused only on genes that are known to encode mediators of inflammatory pathways (including cytokines, antioxidants and xenobiotic metabolising enzymes) limiting the potential of the study to generate new hypotheses regarding exposure-induced response mechanisms. Indeed, of the four significant transcriptional changes observed, two (dysregulation of superoxide dismutase and glutathione peroxidase expression) have been associated with BDE exposure previously [61, 96]. In contrast, evidence that BDE induced expression of β-2 adrenergic receptors (β_2_AR) [95] was novel. Given that PM_2.5_ has been shown to induce IL-6 secretion in macrophages by stimulating β_2_ARs [97], this result may encourage innovative research into ion channel activity during BDE-induced inflammatory responses.

The first genome-wide transcriptomic analysis of BDE-induced toxicity helped resolve the difficulties in inter-study comparison that surrounded previous hypothesis-driven analyses. To detect mechanisms of toxicity that are common to different types of exhaust particulate, Libalova et al. used standardised methods of particle collection and cellular exposure to study global changes in bronchial epithelial cell gene expression following exposure to the organic fractions of exhaust particles produced through combustion of RME (100 and 30% blends), FD or pure NEXBTL (a combination of HVO and waste animal fat) PM. Due to the genome-wide scope of the array (performed using Illumina Human-HT12 v4 Expression BeadChips), the group were able to demonstrate that RME-derived organic matter altered expression of more genes than samples obtained from NEXBTL and that the extent of transcriptomic changes heightened with increasing RME:FD ratios in the parent fuels (from 0 to 100%) after 4 h exposure. Additionally, the experiment captured the transient changes in gene expression profiles during acute responses to particle extracts. After 4 h of exposure, many transcriptomic changes were induced in common by each of the tested PM fractions, influencing antioxidant defences, xenobiotic/ lipid metabolism, suppression of apoptotic stimuli and blood clotting. After 24 h however, far fewer genes and pathways were dysregulated by all particles and a greater number of fuel-specific dysregulated genes were observed (especially for the 30% RME blend). Most of these fuel-specific changes associated with NEXTBL exposure, indicating that PM produced by combustion of this BD altered expression of genes required to regulate cell cycle progression [98].

Similarly, RME emissions were shown to alter the expression of genes required for cell cycle progression in mice [61]. Pathway analysis (using Ingenuity Pathway Analysis software (IPA)) also associated exposure to RME and FD emissions with ‘*metabolic disease, neurological disease, psychological disorders’*. However, as discussed by the authors, IPA analyses depend upon the size of feature lists to estimate network scores and may not provide accurate representations of dysregulated pathways. Indeed, analyses performed with Pathway Studio software offered considerably different results, associating exposure to FD (but not RME) emissions with dysregulation of genes required for DNA repair pathways. This output contrasted with that produced by IPA but was supported by evidence that FDE induced development of acrolein adducts and induction of phosphorylated histone H2AX genotoxicity markers [61], highlighting the importance of selecting appropriate bioinformatic workflows.

It must be noted that molecular responses to particulate insult can occur downstream and independently of the transcriptome. For example, residual oily fly ash, urban dust and volcanic ash directly inhibit the activity of antioxidant enzymes with capacities that reflect their relative toxicity in vitro [64]. Mechanisms such as these would be excluded from characterisations of particle toxicity if transcriptomic screens were studied alone. Additionally, many ambient particulate species induce degradation of oxidised proteins [99] indicating that not all transcriptional events will yield protein-level activity under conditions of particle-induced oxidative stress.

#### Metabolomics and mass-spectrometry based profiling

As the product of upstream molecular events, the metabolome is considered an important source of mechanistically relevant biomarkers for cellular activities that can be explored alongside the transcriptome or proteome. Compared with these analyses however, production of comprehensive metabolomic profiles is challenging. Properties such as polarity or volatility require molecular subsets to be quantified through distinctly optimised platforms and annotation of metabolites in the resulting spectra is far from complete. A multi-platform approach has been used to measure the changes that BDE exposure induces upon the pulmonary metabolome; combining ^1^H nuclear magnetic resonance spectroscopy (^1^H NMR), liquid chromatography-mass spectrometry (LC-MS) and gas chromatography-mass spectrometry (GC-MS) methods to identify markers of BDE toxicity in human bronchial wash (BW) and BALF samples [100]. By profiling distinct molecular subsets, this use of complimentary methods greatly enhanced the number of metabolites that were detected in the samples; a feature that related directly to the number of pathways that were associated with BDE exposure via pathway enrichment analysis. While requiring validation, connections between these pathways and BDE exposure were novel, implicating degradation of cell membrane lipids as a driver of the response alongside alterations in energy metabolism [100]. Importantly, the analysis demonstrated that BDE-induced alterations to pulmonary metabolism were highly compartment specific, with concentrations of individual metabolites differing significantly between the BW and BALF and a stronger response being detectable for peripheral regions of the lungs [100]. Extending this work, Gouveia-Fugueira et al. showed that human BW and BALF samples could be differentiated by their lipid mediator profiles with concentrations of prostaglandin- E2, 12,13-dihydroxy-9Z-octadecenoic acid, and 13-hydroxyoctadecadienoic acid being significantly elevated in BALF following exposure to BDE [101]. Given that these metabolites and inflammatory lipids are the products, substrates, intermediates and regulators of cellular reactions, this evidence provides a sturdy rationale for focusing on responses in the peripheral airways – an assumption that is often made based on estimated deposition patterns for similarly sized particles [102].

#### Future directions

**Fig. 2:**
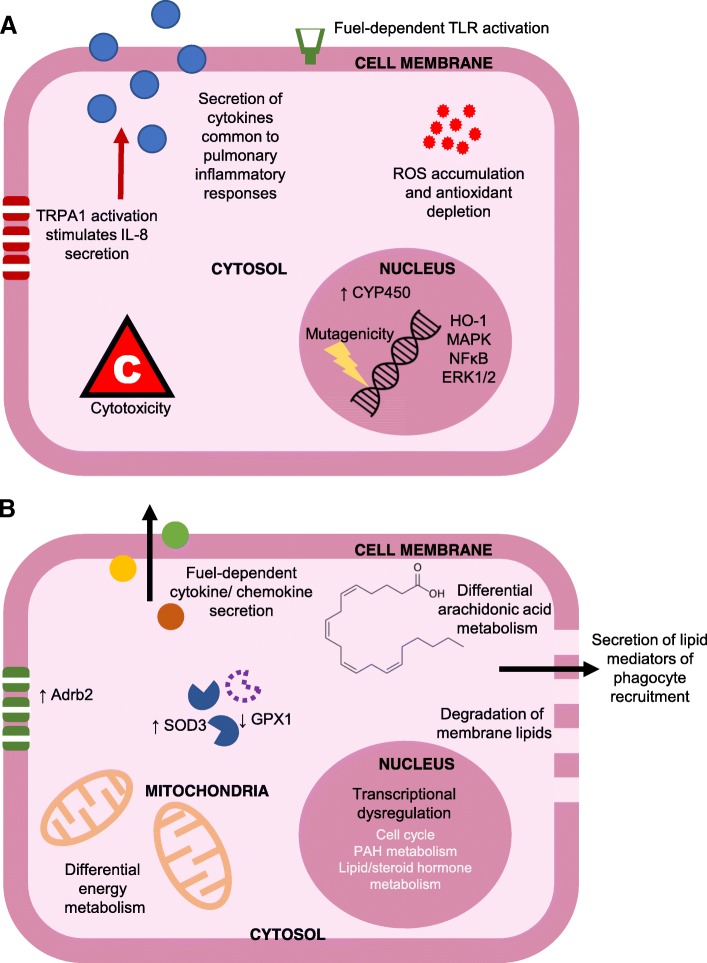
General overview of known molecular and cellular features of pulmonary responses to BDE exposure, including details detected via targeted (**a**) and untargeted (**b**) experimental approaches in samples harvested from human cohorts or in vitro and in vivo models of human and rodent airways

Performance of omics-led experimentation has considerably advanced characterisations of the mechanisms that underlie BDE-induced toxicity (Fig. 2). Indeed, with the large range of platforms available for molecular profiling, it will be possible to identify novel pathways and regulators of toxicity and to make comprehensive comparisons between the molecular interactions that associate with BDE and FDE. As a starting point, expansion of metabonomic studies may be especially useful; as not only do metabolites incorporate the series of molecular events that occurred upstream of their synthesis (including epigenomic control of gene expression, post-translational modifications etc) but, as products of enzymatic reactions, they demonstrate those molecular events which have *already* occurred in samples. Subsequently, genomic, transcriptomic or proteomic data would be greatly useful further along the characterisation process where information regarding the origin of pathway dysregulation is required, though significant computational and statistical challenges exist in bring these large data sets together. Here, additional screens such as epigenetic or microRNA profiling will provide complementary information regarding the mechanisms by which gene transcription or protein synthesis are dysregulated by exposure.

Although availability of human lung tissue is limited by the invasive nature of biopsy, the multi-platform metabolomic screens performed have demonstrated that metabolite markers of pulmonary responses to BDE exposure are detectable outside of the tissue itself [100]. While collection of BALF or BW remains an invasive procedure, urine and plasma (both rich in metabolite species) can be harvested quickly and painlessly, promoting greater participant recruitment for studies. Metabolite biomarkers of inflammatory and oxidative stress responses to cigarette smoke and metallic welding fumes have been detected previously in human urine and blood [103, 104], indicating that these samples have potential as sources of mechanistically relevant biomarkers for pulmonary responses to other particulates. Should similar urinary or blood biomarkers be identified following BDE exposure, adverse outcomes of exposure to the pollutant could be detected and monitored in large human cohorts, enhancing the representability of characterised mechanisms to the population in general and permitting detection or exploration of the response in high susceptibility groups, including those with existing pulmonary conditions where non-critical, invasive procedures are ill advised [105].

Integration of omics approaches to encompass multiple molecular species could expand the number of pathways that are bioinformatically associated with exposure by generating a greater number of novel hypotheses. Indeed, the detection rate of mechanistically relevant pathways resulting from responses to chemotherapeutics was improved by 76% by integrating transcriptomic and metabolomic features during pathway analysis [106]. Considering that the incidence of type I errors increases with the number of variables that are input into a pathway analysis, complementary screens also provide the important function of cross-validation. The observation of a hierarchical proteomic response to FDE-PM exposure for example, has been validated at the transcriptional level by evidence that mid-range concentrations of FDE-PM induce expression of genes that encode pro-inflammatory mediators but not antioxidant enzymes in rat macrophages [107].

Finally, it would be prudent to apply profiling techniques to the exploration of non-classical models of pulmonary exposure, widening our understanding beyond responses of the airway epithelium and innate immune system. For example, possible neuronal responses to BDE exposure were highlighted by transcriptomic analysis of mouse lung (associating RME emission exposure with ‘neuronal disease’ pathways) and the discovery that the SOF of BDE-PM agonises TRPA1 activity [89]. Comprehensive studies of molecular alterations within pulmonary neurons could greatly enhance the breadth of our characterisations of BDE-induced pulmonary toxicity, potentially providing novel explanations of how exposure may trigger respiratory reflexes.

## Conclusions

Introduction of omics strategies to the characterisation of toxicity pathways has greatly progressed our understanding of how human lungs respond to BDE. Moving away from hypotheses inspired by our knowledge of how the lungs respond to FDE, profiling methods have identified mechanistically informative, fuel-specific molecular signatures. Importantly, they have also highlighted mechanisms beyond those that we traditionally associate with particulate exposure, which could encourage exploration into the roles of additional pathways and physiological systems. Such outcomes are difficult to achieve using traditional toxicological testing but would be highly beneficial throughout the field of particulate toxicology. The most recent air quality proposals (such as the UK Clean Air Strategy 2018) pledge to address a range of pollutant sources much wider than the transport combustion engine [108]. Characterisations of the toxicity posed by many of these particles are in their infancy but, with the breadth of information that omics screens offer, policy makers could be provided with a more detailed understanding of how the different particles impact the lungs, and whether some cause more diverse toxicities than others. This information could assist targeting of regulatory efforts to those pollutants that pose the greatest risk to public health and help to prevent unexpected consequences by focusing on new fuels, or engine technologies that may offer solutions to current problems but introduce a new set of problems. The history of air pollution regulation is littered with such examples [109], with the European policies of the mid-1990s promoting the uptake of diesel vehicles being the clearest contemporary example. Embracing the advances in multi-omics technologies, allied to traditional hypothesis-led approaches to address the safety of new fuels presents the opportunity to develop evidence-based pollutant mitigations strategies, rather than simplistic fixes that often fail to consider the health of the population.

## Abbreviations

^1^H NMR: Proton nuclear magnetic resonance spectroscopy
AhR: Aryl-hydrocarbon receptor
BALF: Bronchoalveolar lavage fluid
BDE: Biodiesel exhaust
BDE-PM: Biodiesel exhaust particulate matter
bFGF: Basic fibroblast growth factor
BW: Bronchial wash
CCL: Chemokine (C-C-motif) ligand
CO: Carbon monoxide
CXCL: Chemokine (C-X-motif) ligand
CYP: Cytochrome P450
EGFR: Epidermal growth factor receptor
EPA: Environmental Protection Agency
ERK: Extracellular signal-regulated kinase
FAME: Fatty acid methyl ester
FD: Fossil diesel
FDE: Fossil diesel exhaust
FDE-PM: Fossil diesel exhaust particulate matter
GC-MS: Gas chromatography-mass spectrometry
G-CSF: Granulocyte-colony stimulating factor
HO-1: Heme oxygenase-1
HVO: Hydrogenated vegetable oil
IL: Interleukin
IP-10: Interferon-γ induced protein
IPA: Ingenuity Pathway Analysis
LC-MS: Liquid chromatography-mass spectrometry
LIF: Leukaemia inhibitory factor
MAOK: Mitogen-activated protein kinase
MCP-1: Monocyte chemoattractant protein-1
M-CSF: Macrophage colony stimulating factor
MIP: Macrophage inflammatory protein
NFκB: Nuclear factor kappa-light-chain-enhancer of activated B cells
NIFNγ: Interferon-γ neutralising
NO_x_: Nitrogen oxides
Nrf2: Nuclear receptor factor 2
PAH: Polycyclic aromatic hydrocarbon
PDGF: Platelet-derived growth factor
PM: Particulate matter
PM_0.1_: Ultrafine particulates
RME: Rapeseed methyl ester
SME: Soybean methyl ester
SOF: Soluble organic fraction
TLR: Toll-like receptor
TNF-α: Tumour necrosis factor-α
TRPA1: Transient receptor potential ankyrin 1
VEGF: Vascular endothelial growth factor
WGPM: Waste grease biodiesel exhaust particulate matter
β_2_AR: β-2 adrenergic receptors
